# 1-(4-Bromo­phen­yl)-2-methyl-1*H*-indole-3-carbonitrile

**DOI:** 10.1107/S1600536811034246

**Published:** 2011-08-27

**Authors:** Qiao Yan, Xiuxiang Qi

**Affiliations:** a School of Pharmaceutical Science and Technology, Tianjin University, Tianjin 300072, People’s Republic of China

## Abstract

In the title compound, C_16_H_11_BrN_2_, the dihedral angle between the indole ring system and the phenyl ring is 58.85 (11)°.

## Related literature

For the synthesis of the title compound, see: Du *et al.* (2006 ▶). For its precursor, see: Jin *et al.* (2009 ▶). For related structures, see: Yang *et al.* (2011 ▶); Yan & Qi (2011 ▶).
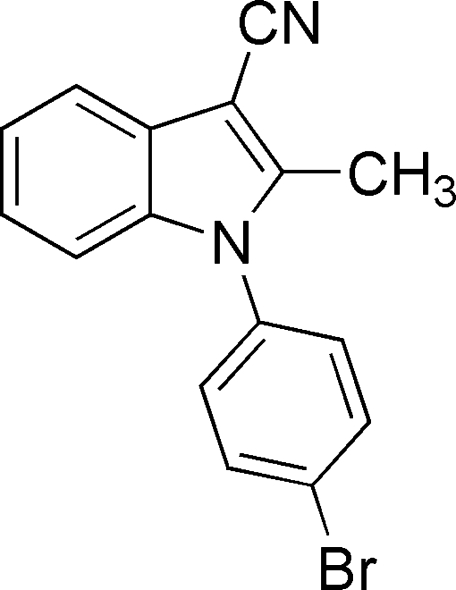


## Experimental

### 

#### Crystal data

C_16_H_11_BrN_2_
*M* *_r_* = 311.18
Monoclinic, 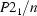

*a* = 9.170 (7) Å
*b* = 8.849 (6) Å
*c* = 16.337 (12) Å
β = 94.415 (15)°
*V* = 1321.7 (16) Å^3^
*Z* = 4
Mo *K*α radiation
μ = 3.10 mm^−1^
*T* = 113 K
0.20 × 0.18 × 0.14 mm

#### Data collection

Rigaku Saturn724 CCD diffractometer
Absorption correction: multi-scan (*CrystalClear*; Rigaku, 2009 ▶) *T*_min_ = 0.576, *T*_max_ = 0.671
12992 measured reflections
3147 independent reflections
2309 reflections with *I* > 2σ(*I*)
*R*_int_ = 0.052

#### Refinement

*R*[*F* ^2^ > 2σ(*F* ^2^)] = 0.042
*wR*(*F* ^2^) = 0.106
*S* = 1.00
3147 reflections
173 parameters
H-atom parameters constrained
Δρ_max_ = 0.90 e Å^−3^
Δρ_min_ = −0.73 e Å^−3^

### 

Data collection: *CrystalClear-SM Expert* (Rigaku, 2009 ▶); cell refinement: *CrystalClear-SM Expert*; data reduction: *CrystalClear-SM Expert*; program(s) used to solve structure: *SHELXS97* (Sheldrick, 2008 ▶); program(s) used to refine structure: *SHELXL97* (Sheldrick, 2008 ▶); molecular graphics: *CrystalStructure* (Rigaku, 2009 ▶); software used to prepare material for publication: *CrystalStructure*.

## Supplementary Material

Crystal structure: contains datablock(s) global, I. DOI: 10.1107/S1600536811034246/hb6373sup1.cif

Structure factors: contains datablock(s) I. DOI: 10.1107/S1600536811034246/hb6373Isup2.hkl

Supplementary material file. DOI: 10.1107/S1600536811034246/hb6373Isup3.cml

Additional supplementary materials:  crystallographic information; 3D view; checkCIF report

## Figures and Tables

**Fig. 1.**
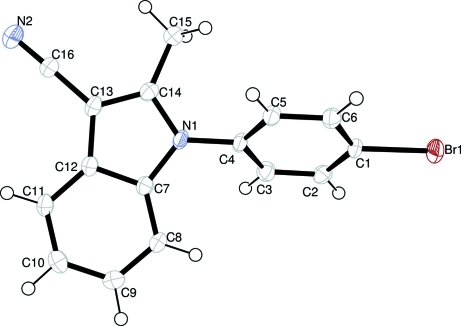
The molecular structure of (I) with 50% probability displacement ellipsoids.

## supplementary crystallographic information

### Comment

In our continuing investigation of indole derivatives, herein, we reported the title compound (I). In the molecular structure,(Fig. 1), the indole ring is essentially planar with a dihedral angle of 0.95 (16)% between its pyrrole ring and fused benzene ring, similar to that [0.85 (6)°] of the 1-(2-chlorophenyl)- 6-fluoro-2-methyl-1*H*-indole-3-carbonitrile (Yang *et al.*, 2011), but less than that [2.66 (6)°] of our previously reported 1-(4-methoxyphenyl)-2-methyl-1*H*-indole-3-carbonitrile (Yan & Qi, 2011).

The indole ring constructs an angle of 58.85 (11) ° with the bromobenzene ring, similar to that [58.41 (4)°] reported by our group (Yan & Qi, 2011), but less than that [80.91 (5)°] reported by Yang *et al.* (2011). All the difference might be attributed to the steric substituent on the *N*-phenyl motif.

In the crystal packing, no significant π-π stacking interaction and C—H···π interaction were detected, unlike those reported in its anolog 1-(4-methoxyphenyl) -2-methyl-1*H*-indole-3-carbonitrile.

### Experimental

The title compound was prepared according to the method of the literature (Du, *et al.*, 2006). Colourless prisms of (I) were grown from a mixture of ethyl actate and petroleum ether.

### Refinement

All H atoms were positioned geometrically (C—H = 0.95 and 0.98 Å)and refined as riding with *U*_iso_(H) = 1.2*U*_eq_(CH) or 1.5*U*_eq_(CH_3_).

### Crystal data

**Table:**

C_16_H_11_BrN_2_	*F*(000) = 624
*M**_r_* = 311.18	*D*_x_ = 1.564 Mg m^−^^3^
Monoclinic, *P*2_1_/*n*	Mo *K*α radiation, λ = 0.71073 Å
Hall symbol: -P 2yn	Cell parameters from 4781 reflections
*a* = 9.170 (7) Å	θ = 2.2–27.9°
*b* = 8.849 (6) Å	µ = 3.10 mm^−^^1^
*c* = 16.337 (12) Å	*T* = 113 K
β = 94.415 (15)°	Prism, colorless
*V* = 1321.7 (16) Å^3^	0.20 × 0.18 × 0.14 mm
*Z* = 4	

### Data collection

**Table:**

Rigaku Saturn724 CCD diffractometer	3147 independent reflections
Radiation source: rotating anode	2309 reflections with *I* > 2σ(*I*)
multilayer	*R*_int_ = 0.052
Detector resolution: 14.22 pixels mm^-1^	θ_max_ = 27.9°, θ_min_ = 2.5°
ω and φ scans	*h* = −12→11
Absorption correction: multi-scan (*CrystalClear*; Rigaku, 2009)	*k* = −11→11
*T*_min_ = 0.576, *T*_max_ = 0.671	*l* = −21→21
12992 measured reflections	

### Refinement

**Table:**

Refinement on *F*^2^	Primary atom site location: structure-invariant direct methods
Least-squares matrix: full	Secondary atom site location: difference Fourier map
*R*[*F*^2^ > 2σ(*F*^2^)] = 0.042	Hydrogen site location: inferred from neighbouring sites
*wR*(*F*^2^) = 0.106	H-atom parameters constrained
*S* = 1.00	*w* = 1/[σ^2^(*F*_o_^2^) + (0.0495*P*)^2^] where *P* = (*F*_o_^2^ + 2*F*_c_^2^)/3
3147 reflections	(Δ/σ)_max_ < 0.001
173 parameters	Δρ_max_ = 0.90 e Å^−^^3^
0 restraints	Δρ_min_ = −0.73 e Å^−^^3^

### Special details

**Table:**

Geometry. All e.s.d.'s (except the e.s.d. in the dihedral angle between two l.s. planes) are estimated using the full covariance matrix. The cell e.s.d.'s are taken into account individually in the estimation of e.s.d.'s in distances, angles and torsion angles; correlations between e.s.d.'s in cell parameters are only used when they are defined by crystal symmetry. An approximate (isotropic) treatment of cell e.s.d.'s is used for estimating e.s.d.'s involving l.s. planes.
Refinement. Refinement of *F*^2^ against ALL reflections. The weighted *R*-factor *wR* and goodness of fit *S* are based on *F*^2^, conventional *R*-factors *R* are based on *F*, with *F* set to zero for negative *F*^2^. The threshold expression of *F*^2^ > σ(*F*^2^) is used only for calculating *R*-factors(gt) *etc*. and is not relevant to the choice of reflections for refinement. *R*-factors based on *F*^2^ are statistically about twice as large as those based on *F*, and *R*- factors based on ALL data will be even larger.

### Fractional atomic coordinates and isotropic or equivalent isotropic displacement parameters (Å^2^)

**Table:**

	*x*	*y*	*z*	*U*_iso_*/*U*_eq_	
Br1	1.31610 (4)	0.01069 (4)	0.08227 (2)	0.03260 (14)	
N1	0.8376 (3)	0.4726 (3)	0.12974 (16)	0.0204 (6)	
N2	0.3390 (3)	0.6165 (3)	0.11113 (18)	0.0341 (7)	
C1	1.1681 (3)	0.1574 (3)	0.0968 (2)	0.0229 (7)	
C2	1.1611 (4)	0.2252 (3)	0.1735 (2)	0.0248 (7)	
H2	1.2302	0.2002	0.2178	0.030*	
C3	1.0509 (4)	0.3301 (3)	0.18380 (19)	0.0243 (7)	
H3	1.0444	0.3783	0.2354	0.029*	
C4	0.9508 (3)	0.3642 (3)	0.11868 (19)	0.0205 (6)	
C5	0.9610 (3)	0.2969 (3)	0.04233 (19)	0.0215 (7)	
H5	0.8934	0.3230	−0.0024	0.026*	
C6	1.0702 (3)	0.1913 (3)	0.0317 (2)	0.0236 (7)	
H6	1.0770	0.1434	−0.0199	0.028*	
C7	0.8620 (3)	0.6253 (3)	0.15065 (18)	0.0198 (6)	
C8	0.9938 (3)	0.6992 (3)	0.17217 (18)	0.0225 (7)	
H8	1.0846	0.6470	0.1749	0.027*	
C9	0.9861 (4)	0.8520 (3)	0.18934 (19)	0.0250 (7)	
H9	1.0732	0.9057	0.2058	0.030*	
C10	0.8526 (4)	0.9285 (4)	0.18296 (19)	0.0264 (7)	
H10	0.8513	1.0340	0.1938	0.032*	
C11	0.7227 (4)	0.8555 (3)	0.16141 (18)	0.0236 (7)	
H11	0.6327	0.9093	0.1574	0.028*	
C12	0.7265 (3)	0.6991 (3)	0.14552 (18)	0.0206 (7)	
C13	0.6191 (3)	0.5860 (3)	0.12233 (18)	0.0219 (7)	
C14	0.6892 (3)	0.4497 (3)	0.11365 (19)	0.0218 (7)	
C15	0.6229 (4)	0.2960 (3)	0.0985 (2)	0.0283 (8)	
H15A	0.6327	0.2370	0.1494	0.042*	
H15B	0.6736	0.2440	0.0560	0.042*	
H15C	0.5190	0.3068	0.0803	0.042*	
C16	0.4642 (4)	0.6019 (3)	0.11543 (19)	0.0245 (7)	

### Atomic displacement parameters (Å^2^)

**Table:**

	*U*^11^	*U*^22^	*U*^33^	*U*^12^	*U*^13^	*U*^23^
Br1	0.02374 (19)	0.0305 (2)	0.0430 (2)	0.01071 (15)	−0.00149 (15)	−0.00554 (15)
N1	0.0142 (12)	0.0235 (14)	0.0236 (14)	0.0045 (10)	0.0013 (11)	−0.0011 (10)
N2	0.0214 (16)	0.0380 (17)	0.0430 (18)	0.0044 (13)	0.0025 (14)	−0.0032 (13)
C1	0.0152 (15)	0.0197 (15)	0.0338 (18)	0.0052 (13)	0.0009 (13)	−0.0005 (13)
C2	0.0201 (16)	0.0257 (17)	0.0273 (17)	0.0019 (14)	−0.0059 (14)	0.0001 (13)
C3	0.0238 (17)	0.0261 (16)	0.0229 (17)	0.0023 (14)	0.0007 (14)	−0.0023 (12)
C4	0.0144 (15)	0.0214 (16)	0.0260 (16)	0.0040 (12)	0.0029 (13)	0.0004 (12)
C5	0.0185 (16)	0.0249 (16)	0.0211 (16)	0.0032 (13)	0.0018 (13)	−0.0012 (12)
C6	0.0219 (17)	0.0252 (16)	0.0238 (17)	0.0020 (13)	0.0017 (14)	−0.0027 (12)
C7	0.0184 (16)	0.0219 (16)	0.0194 (15)	0.0016 (13)	0.0031 (13)	−0.0001 (12)
C8	0.0164 (16)	0.0262 (17)	0.0249 (17)	0.0029 (13)	0.0021 (13)	0.0000 (12)
C9	0.0222 (18)	0.0271 (17)	0.0254 (17)	−0.0042 (14)	0.0004 (14)	0.0013 (13)
C10	0.0339 (19)	0.0220 (16)	0.0236 (17)	0.0031 (15)	0.0045 (15)	−0.0014 (12)
C11	0.0215 (17)	0.0245 (17)	0.0254 (17)	0.0059 (13)	0.0059 (14)	0.0000 (12)
C12	0.0190 (16)	0.0221 (16)	0.0211 (16)	0.0035 (13)	0.0039 (13)	0.0011 (12)
C13	0.0165 (15)	0.0259 (16)	0.0237 (16)	0.0034 (13)	0.0044 (13)	0.0022 (12)
C14	0.0180 (16)	0.0261 (16)	0.0216 (16)	−0.0003 (13)	0.0024 (13)	−0.0003 (12)
C15	0.0212 (17)	0.0278 (18)	0.036 (2)	0.0005 (14)	0.0003 (15)	−0.0058 (14)
C16	0.0201 (17)	0.0251 (17)	0.0283 (17)	0.0010 (14)	0.0019 (14)	−0.0014 (13)

### Geometric parameters (Å, °)

**Table:**

Br1—C1	1.906 (3)	C7—C12	1.401 (4)
N1—C14	1.381 (4)	C8—C9	1.384 (4)
N1—C7	1.408 (4)	C8—H8	0.9500
N1—C4	1.435 (4)	C9—C10	1.396 (4)
N2—C16	1.152 (4)	C9—H9	0.9500
C1—C6	1.371 (4)	C10—C11	1.377 (4)
C1—C2	1.396 (4)	C10—H10	0.9500
C2—C3	1.393 (4)	C11—C12	1.410 (4)
C2—H2	0.9500	C11—H11	0.9500
C3—C4	1.384 (4)	C12—C13	1.434 (4)
C3—H3	0.9500	C13—C14	1.379 (4)
C4—C5	1.392 (4)	C13—C16	1.423 (4)
C5—C6	1.391 (4)	C14—C15	1.503 (4)
C5—H5	0.9500	C15—H15A	0.9800
C6—H6	0.9500	C15—H15B	0.9800
C7—C8	1.395 (4)	C15—H15C	0.9800
			
C14—N1—C7	108.8 (2)	C7—C8—H8	121.6
C14—N1—C4	126.1 (3)	C8—C9—C10	121.1 (3)
C7—N1—C4	124.7 (3)	C8—C9—H9	119.4
C6—C1—C2	122.1 (3)	C10—C9—H9	119.4
C6—C1—Br1	118.8 (2)	C11—C10—C9	121.9 (3)
C2—C1—Br1	119.1 (2)	C11—C10—H10	119.1
C3—C2—C1	118.6 (3)	C9—C10—H10	119.1
C3—C2—H2	120.7	C10—C11—C12	118.3 (3)
C1—C2—H2	120.7	C10—C11—H11	120.8
C4—C3—C2	119.8 (3)	C12—C11—H11	120.8
C4—C3—H3	120.1	C7—C12—C11	118.7 (3)
C2—C3—H3	120.1	C7—C12—C13	106.2 (3)
C3—C4—C5	120.6 (3)	C11—C12—C13	135.1 (3)
C3—C4—N1	119.5 (3)	C14—C13—C16	123.2 (3)
C5—C4—N1	119.9 (3)	C14—C13—C12	108.8 (3)
C6—C5—C4	119.9 (3)	C16—C13—C12	127.8 (3)
C6—C5—H5	120.0	C13—C14—N1	108.2 (3)
C4—C5—H5	120.0	C13—C14—C15	128.5 (3)
C1—C6—C5	119.0 (3)	N1—C14—C15	123.0 (3)
C1—C6—H6	120.5	C14—C15—H15A	109.5
C5—C6—H6	120.5	C14—C15—H15B	109.5
C8—C7—C12	123.0 (3)	H15A—C15—H15B	109.5
C8—C7—N1	129.0 (3)	C14—C15—H15C	109.5
C12—C7—N1	108.0 (3)	H15A—C15—H15C	109.5
C9—C8—C7	116.9 (3)	H15B—C15—H15C	109.5
C9—C8—H8	121.6	N2—C16—C13	178.7 (4)
			
C6—C1—C2—C3	0.1 (5)	C9—C10—C11—C12	0.0 (5)
Br1—C1—C2—C3	−178.9 (2)	C8—C7—C12—C11	1.1 (5)
C1—C2—C3—C4	0.4 (5)	N1—C7—C12—C11	−178.1 (3)
C2—C3—C4—C5	−1.4 (5)	C8—C7—C12—C13	−179.8 (3)
C2—C3—C4—N1	−179.9 (3)	N1—C7—C12—C13	1.0 (3)
C14—N1—C4—C3	−125.7 (3)	C10—C11—C12—C7	−1.3 (4)
C7—N1—C4—C3	61.5 (4)	C10—C11—C12—C13	179.9 (3)
C14—N1—C4—C5	55.8 (4)	C7—C12—C13—C14	−0.3 (3)
C7—N1—C4—C5	−117.1 (3)	C11—C12—C13—C14	178.6 (3)
C3—C4—C5—C6	1.7 (5)	C7—C12—C13—C16	174.7 (3)
N1—C4—C5—C6	−179.7 (3)	C11—C12—C13—C16	−6.4 (6)
C2—C1—C6—C5	0.2 (5)	C16—C13—C14—N1	−175.8 (3)
Br1—C1—C6—C5	179.2 (2)	C12—C13—C14—N1	−0.6 (3)
C4—C5—C6—C1	−1.1 (5)	C16—C13—C14—C15	−2.0 (5)
C14—N1—C7—C8	179.4 (3)	C12—C13—C14—C15	173.2 (3)
C4—N1—C7—C8	−6.7 (5)	C7—N1—C14—C13	1.2 (3)
C14—N1—C7—C12	−1.4 (3)	C4—N1—C14—C13	−172.6 (3)
C4—N1—C7—C12	172.5 (3)	C7—N1—C14—C15	−173.0 (3)
C12—C7—C8—C9	0.4 (5)	C4—N1—C14—C15	13.2 (5)
N1—C7—C8—C9	179.5 (3)	C14—C13—C16—N2	136 (18)
C7—C8—C9—C10	−1.8 (5)	C12—C13—C16—N2	−38 (18)
C8—C9—C10—C11	1.6 (5)		
